# *Pimelea* and Its Toxicity: A Survey of Landholder Experiences and Management Practices

**DOI:** 10.3390/toxins17080393

**Published:** 2025-08-06

**Authors:** Rashid Saleem, Shane Campbell, Mary T. Fletcher, Sundaravelpandian Kalaipandian, Steve W. Adkins

**Affiliations:** 1 School of Agriculture and Food Sustainability, The University of Queensland, Gatton, QLD 4343, Australia; shane.campbell@uq.edu.au (S.C.); s.adkins@uq.edu.au (S.W.A.); 2 Queensland Alliance for Agriculture and Food Innovation, The University of Queensland, Coopers Plains, Brisbane, QLD 4108, Australia; mary.fletcher@uq.edu.au; 3 Department of Bioengineering, Saveetha Institute of Medical and Technical Sciences (SIMATS), Saveetha School of Engineering, Chennai 602105, India

**Keywords:** *Pimelea* poisoning, impact on livestock, management strategies, livestock producers: general awareness, *Pimelea* impact survey

## Abstract

*Pimelea* is one of the highly toxic plants in Australia, particularly affecting cattle. It contains simplexin, a potent toxin that can cause *Pimelea* poisoning (St. George Disease) in livestock. A survey was conducted to assess the current impact of *Pimelea* on livestock production, pasture systems, and financial losses among agricultural producers. In addition, information was also sought about the environmental conditions that facilitate its growth and the effectiveness of existing management strategies. The survey responses were obtained from producers affected by *Pimelea* across nine different Local Government Areas, through three States, viz., Queensland, New South Wales, and South Australia. *Pimelea* was reported to significantly affect animal production, with 97% of producers surveyed acknowledging its detrimental effects. Among livestock, cattle were the most severely affected (94%), when compared to sheep (13%), goats (3%), and horses (3%). The presence of *Pimelea* was mostly observed in spring (65%) and winter (48%), although 29% of respondents indicated that it could be present all year-round under favorable rainfall conditions. Germination was associated with light to moderate rainfall (52%), while only 24% linked it to heavy rainfall. *Pimelea simplex* F. Muell. was the most frequently encountered species (71%), followed by *Pimelea trichostachya* Lindl. (26%). Infestations were reported to occur annually by 47% of producers, with 41% noting occurrences every 2 to 5 years. Financially, producers estimated average annual losses of AUD 67,000, with 50% reporting an average of 26 cattle deaths per year, reaching up to 105 deaths in severe years. Some producers were spending up to AUD 2100 per annum to manage *Pimelea*. While chemical and physical controls were commonly employed, integrating competitive pastures and alternative livestock, such as sheep and goats, was considered as a potential management strategy. This study reiterates the need for further research on sustainable pasture management practices to reduce *Pimelea*-related risks to livestock and agricultural production systems.

## 1. Introduction

*Pimelea* poisoning poses a significant economic threat to the Australian livestock industry, primarily due to the toxicity of three noxious species: *Pimelea trichostachya*, *Pimelea simplex*, and *Pimelea elongata* Threlfall [1]. *Pimelea* species are regarded as annuals [1] that favorably grow in the Australian winter–spring period [2]. The diterpenoid orthoester simplexin has been identified as the putative toxin present in these species of *Pimelea* [3,4], including *P. elongata*, *P. simplex* (both subspecies *simplex* and *continua*), and *P. trichostachya* [5,6,7]. These species can cause severe health issues in livestock, particularly cattle (*Bos taurus* L. and *Bos indicus* L.) [8]. The ingestion of *Pimelea* plants can result in cattle fatalities or leave surviving animals weakened, significantly reducing their productivity [7]. Simplexin, the main toxin in *Pimelea* species, triggers pimelea poisoning in cattle by activating protein kinase C. This causes blood vessel constriction between the lungs and heart, leading to increased pressure, fluid buildup in the chest, heart enlargement, and subcutaneous oedema [7]. Based on the published literature, simplexin is found at the highest concentrations in seeds and flowers, with moderate levels in leaves and lower levels in stems. Toxicity can persist in dry plant material and residues, including senesced stems and soil-borne seeds, indicating its prolonged residence time and posing a risk to grazing livestock even after the plant has died back [7]. The reduction in cattle health and productivity due to *Pimelea* poisoning often leads to a decrease in pasture carrying capacity, affecting the overall sustainability and profitability of affected agricultural systems.

The presence of *Pimelea* populations in the field is often unpredictable and sporadic, making it challenging for livestock producers to anticipate and manage infestations effectively [7]. The *Pimelea* plant genus is native to Australia, New Zealand, Timor, and Lord Howe Island [9], and it comprises 140 species, with about 90 species found in inland Australia [10]. Ecologically, these plants are often found in disturbed rangelands, open grasslands, and degraded pastures [7]. This sporadic appearance and distribution highlight the need for a comprehensive understanding of the seed ecology of the toxic *Pimelea* species. By studying the conditions under which these plants germinate, grow, and mature, researchers can better predict when and where *Pimelea* will appear in the field. This knowledge is essential for developing proactive and cost-effective management strategies that minimize the risk of livestock exposure.

Effective management is particularly important because some methods do not address the underlying issue of toxic plant residues being left in the field. Even after plants have been removed, the remaining toxic stems can still pose a significant threat to livestock, as they can be ingested by grazing animals [7]. Therefore, management strategies must not only focus on killing the plants but also on preventing the toxic residues from remaining in the field. By understanding the seed biology and germination patterns of *Pimelea*, researchers can help develop strategies that break the lifecycle of these toxic plants and reduce their impact on agricultural productivity.

Pest animals, diseases, and weeds contribute to both direct and indirect losses in the livestock industry. Direct losses primarily involve reduced production, such as a diminished forage supply or livestock poisoning, while indirect losses result from increased management costs, including expenses for agistment (grazing land rental) and other control measures. Among these threats, plant poisoning is becoming an increasingly significant concern for the livestock sector. In this regard, *Pimelea* poisoning is one of the most serious threats to cattle in the drier regions of Australia. It is most reported in the arid and semi-arid areas of western Queensland (QLD), western New South Wales (NSW), and northern South Australia (SA) [11]. The sporadic nature of the appearance of *Pimelea* in the field in these regions complicates management efforts, with affected livestock often experiencing acute poisoning leading to mortality or, in surviving animals, long-term productivity losses. As a result, the economic burden on livestock producers can be considerable, emphasizing the need for improved management strategies and preventive measures to mitigate the impact of *Pimelea* and other plant poisons on the livestock industry.

A survey conducted in 2017, involving 30 producers from the Balonne and Maranoa Shires in QLD, revealed a significant economic impact on the livestock industry due to *Pimelea* poisoning. On average, each property reported a financial loss of AUD 45,000 annually, with an average of 40 cattle deaths per year across all properties. In some years, losses reached as high as 250 heads due to *Pimelea* poisoning [12]. Beyond the financial costs, producers reported that they also experienced considerable mental stress as they were forced to move their stock to *Pimelea*-free pastures and cull heavily affected animals. However, this 2017 survey primarily focused on the financial impacts of *Pimelea* poisoning and did not explore other crucial aspects, such as appearance of *Pimelea* in the field, its seedbank ecology, or the specific triggers involved in its germination response.

Given the growing community awareness of this plant, the ineffective management strategies being employed, and the time elapsed since the last survey, it was deemed an opportune moment to conduct a follow-up survey. This new survey aimed to gather more comprehensive information about *Pimelea*, including its general awareness, appearance in the field, associated impacts, and the current management practices employed by producers. The insights from this survey would be invaluable for enhancing integrated weed management (IWM) strategies for *Pimelea*, addressing existing knowledge gaps, and ultimately helping the industry better manage toxic *Pimelea* species. To achieve this, a questionnaire-based, cross-sectional survey was conducted on livestock producers in inland areas of Queensland, New South Wales, and South Australia.

## 2. Results

Survey responses were collected from producers impacted by *Pimelea* across ten Local Government Areas (LGAs) spanning three states: QLD, NSW, and SA (Table 1).

### 2.1. General Awareness of Pimelea Species and Their Habitat

Nearly 91% of producers were familiar with various *Pimelea* species; however, not all were able to identify the plant at all growth stages, including seedling, immature, and mature stages (Table 2). Notably, 6% of producers admitted that they could not identify *Pimelea* at all, and only 37% reported being able to recognize it at the seedling stage. Identification improved as the plant matured, reaching its highest recognition rate at the flowering stage (72%). Once the plants had died and only the dead stems remained, confidence in identification decreased, with just 56% of producers feeling certain they could recognize *Pimelea* at this stage (Table 2).

Regarding the specific species observed, 71% of producers reported that *P. simplex* was the most encountered species, followed by *P. trichostachya* (26%) and then *P. elongata* (19%). In terms of frequency, 47% of producers indicated that infestations occurred on their properties very often (annually), while 41% noted occurrences often (every 2 to 5 years) (Table 2).

Producers reported that the most common season for *Pimelea* presence on their properties was spring (65%), followed by winter (48%). Additionally, 29% of producers noted that *Pimelea* could be found year-round, provided that there was sufficient rainfall to support seedling emergence and establishment (Table 2).

In terms of how *Pimelea* spread to their properties, 69% of producers believed that its fruit or seeds were carried by wind, while 50% cited animal movement, and 44% mentioned water movement as the likely causes of dispersal. When it came to preferred growth conditions, 46% of producers observed that *Pimelea* thrived on heavily grazed lands, while 50% indicated a preference for light red, sandy loams, and red heavy clay soils. Soils with low fertility were also identified as favorable by 30% of producers. (Table 2).

### 2.2. Impact on Pasture Productivity

Among the producers, 75% identified beef production as their primary activity, followed by sheep production (56%), goat production (47%), and crop production (9%) (Table 3). Of those with *Pimelea* on their properties, 22% indicated that it had arrived within the last 5 to 10 years, while 13% reported its arrival within the past 5 years. However, 31% of producers noted that *Pimelea* had been present on their land for at least 30 years. Regarding the severity of infestations, 31% considered *Pimelea* to be a minor problem, 34% regarded it as a moderate issue, and 28% classified it as a major concern. The extent of infestations varied, with 40% of producers reporting that *Pimelea* affected 0 to 10% of their land, 20% indicating 10 to 20% coverage, and 17% highlighting that >50% of their property was infested (Table 3).

The majority of producers (77%) reported *Pimelea* to be primarily found in rangelands or grazing areas, while 55% mentioned its presence along roads and tracks. The density of infestations was most frequently described as moderate (≥10 plants m^−2^) by 31% of producers, while 28% indicated an average density of 5 to 10 plants m^−2^ (Table 3).

While 19% of producers noted negative effects on pasture production, no impacts on crops were reported. Among animals, cattle were most severely affected, as reported by 94% of producers, followed by sheep (13%), goats (3%), and horses (3%) (Table 3). Regarding germination triggers, 52% of producers indicated that light, low, or moderate rainfall events could stimulate *Pimelea* germination, while only 24% believed that high rainfall events were necessary. Other factors such as soil disturbance (caused by activities like track grading) and a moderate increase in temperature were identified as germination triggers by 31% and 24% of producers, respectively (Table 3).

### 2.3. The Approaches Used to Manage Pimelea

The management of *Pimelea* remains a significant challenge for many landholders, with 65% of producers reporting that they were not actively managing *Pimelea* (Table 4). Among the 33% who implemented management, the control strategies used were slashing, cutting, or mowing (12%), hand-pulling (12%), and the planting of forage or competitive pasture crops (6%). Cultivation was unanimously deemed the least effective control method (Table 4).

When asked about the difficulty of managing *Pimelea*, 50% of producers described it as difficult, 30% found it very difficult, and 17% believed it could be managed effectively under specific conditions. On average, producers spent AUD 2100 annually on *Pimelea* management. Herbicide use was reported by 29% of producers as a “moderate” or “highly successful” method, with products such as 2,4-D Amine 625, Glyphosate, Dicamba, Grazon, Amine 700, and Graslan being the most used, while 14% found herbicides relatively ineffective (Table 4).

Grazing strategies also played a role in *Pimelea* management, with 59% of producers believing that spelling affected paddocks from cattle was beneficial and 35% opting to graze sheep or goats in *Pimelea*-infested areas. Competitive pastures were less commonly utilized, with 77% of producers not planting specific species to suppress *Pimelea* growth. However, 23% reported using plants such as sorghum (*Sorghum bicolor* Monech.), stylo (*Stylosanthes guianensis* (Aubl.) Sw.), buffel grass (*Pennisetum ciliare* (L.) Link), and lablab (*Lablab purpureus* (L.) Sweet). Despite these efforts, the effectiveness of management strategies varies widely, highlighting the ongoing difficulty of managing *Pimelea* using the current practices (Table 4). Financial losses due to *Pimelea* were substantial, with 43% of producers estimating an average annual loss of AUD 67,000. Additionally, 50% of producers reported an average of 26 cattle deaths per year due to *Pimelea* poisoning, with some herds losing up to 105 cattle in particularly severe years (Table 4).

## 3. Discussion

The findings of this survey provide critical insights into the challenges posed by toxic *Pimelea* species to livestock production and pasture systems across Queensland (QLD), New South Wales (NSW), and South Australia (SA). This study highlights the cyclical nature of *Pimelea* infestations, its adverse effects on livestock, and the varying perceptions of its severity among producers. While progress has been made in raising awareness about *Pimelea* and its management, significant gaps remain in effective control strategies, particularly for large-scale agricultural systems. Below, we discuss key findings, their implications, and potential pathways for improving *Pimelea* management, supported by references to previous studies.

### 3.1. Identification and Perception of the Problem

The survey revealed that 91% of producers could identify *Pimelea* at some stage of its life cycle, with mature flowering plants being the easiest to recognize (72%) and seedlings the hardest (37%). This aligns with previous studies indicating that plants are often most easily identified during their reproductive or maturity stages [7]. However, early identification is crucial for effective management, as it allows for timely intervention before *Pimelea* reaches reproductive maturity and replenishes the soil seed bank [13]. Increasing awareness of early growth stages through targeted education programs could enhance management outcomes. Perceptions of the severity of *Pimelea* varied significantly among producers. Those experiencing long-term infestations were more likely to view it as a major issue, reflecting its cumulative economic impact on livestock production. Regional variations in infestation severity highlight the need for localized management strategies that consider specific environmental and agricultural contexts [14].

### 3.2. Seasonal Patterns and Germination Triggers

The survey’s findings are consistent with established research on *Pimelea*, highlighting its seasonal growth patterns and environmental preferences. *Pimelea* infestations are most common during winter and spring, as previously documented by Cunningham [2]. This aligns with the plant’s classification as an annual species [15], which thrives in cooler winter conditions. In addition, flowering typically occurs during spring [2], aligning with its growth patterns as an annual species. Dry summer conditions have been observed to facilitate the establishment of *Pimelea*, as noted by Henry [16]. Additionally, winter months that follow hot, dry summers appear to create favorable conditions for *Pimelea* growth and proliferation, as highlighted by [17]. These patterns underscore the importance of seasonal and climatic factors in the growth and spread of *Pimelea*. However, instances of germination during summer and autumn, particularly after light to moderate rainfall, suggest that *Pimelea* can emerge under a wider range of conditions than previously thought. Similarly, Fletcher [7] reported that late summer or autumn rainfall (20–30 mm or more) often triggers germination, with significant recruitment occurring in soil depressions during subsequent dry periods. A recent study on *P. trichostachya* revealed that seeds germinate optimally at 25/15 °C, with light enhancing but not being essential for germination. The seeds showed remarkable adaptability, germinating under water-limited conditions and across a wide pH range (4–8). However, seedling emergence was significantly inhibited when seeds were buried deeper than 3 cm, emphasizing the importance of shallow burial for successful establishment. These findings highlight the species’ resilience and suggest that management strategies, such as deep tillage, could be effective in controlling its spread in affected areas [18].

*Pimelea* tend to thrive in heavily grazed lands and specific soil types (e.g., light red, sandy loams or red, heavy clay soils). Overgrazing creates bare patches ideal for *Pimelea* establishment, emphasizing the importance of rotational grazing and maintaining pasture cover as key management strategies [7].

Furthermore, there is substantial circumstantial evidence indicating that *Pimelea* densities are often highest in areas where pasture condition is poorest [2]. This suggests a possible ecological relationship between degraded pastures and the proliferation of *Pimelea*, potentially due to reduced competition from other vegetation or the plant’s ability to exploit disturbed or nutrient-poor soils. This highlights the importance of maintaining healthy pasture conditions to mitigate the spread and impact of *Pimelea* infestations.

### 3.3. Livestock Poisoning and Economic Impact

The survey results indicate that cattle are the most severely affected by *Pimelea* poisoning, with 94% of producers reporting significant impacts. This is followed by sheep (13%), goats (3%), and horses (3%), which appear to be less susceptible or affected to a much lesser extent. The pronounced impact on cattle highlights their heightened vulnerability to *Pimelea* toxicity compared to other livestock species. This disparity may be attributed to differences in grazing behavior, physiological sensitivity, or metabolic responses to the toxic compounds in *Pimelea*. The toxin simplexin, present in all plant parts, poses a year-round risk, as even dead plant material retains its toxicity for months [7]. Many weeks after feeding on the plants, disease symptoms and death of animals can still occur. Profuse diarrhea with loss of condition, roughening of the coat, and jugular distension and pulsation are the early symptoms [19,20]. The sporadic nature of poisoning outbreaks makes management challenging. These findings emphasize the need for targeted management strategies to protect cattle, which are the most at-risk livestock group, while also considering the potential, albeit lower, risks to sheep, goats, and horses.

Economic losses due to *Pimelea* poisoning are substantial, estimated at AUD 50 million annually, including production losses, stock deaths, control measures, and additional management efforts. Stressors such as calving can exacerbate poisoning effects, sometimes leading to sudden cattle deaths [21]. This underscores the need for integrated management strategies addressing both biological and economic aspects of the problem. The current survey highlights an even more concerning trend in the impact of *Pimelea* poisoning on livestock producers. In addition to the significant financial losses, which have risen by 48.89% from AUD 45,000 to AUD 67,000 per producer annually, the survey reveals that 50% of producers reported an average of 26 cattle deaths per year due to *Pimelea* poisoning. In particularly severe years, some herds experienced losses of up to 105 cattle. The reported average annual cost of AUD 2100 for managing *Pimelea* highlights the ongoing financial burden faced by producers, even before accounting for livestock losses. This figure likely includes expenses related to herbicide application, pasture management, supplementary feeding to reduce grazing in infested areas, and labor costs. Despite this investment, the majority of respondents still rated *Pimelea* as difficult or very difficult to manage, suggesting that current control measures may be only partially effective or unsustainable over time. The AUD 2100 cost represents a recurring strain on farm budgets and, when coupled with substantial cattle mortality and rising financial losses, underscores the need for more cost-effective and impactful management strategies tailored to diverse production systems and environmental conditions. These findings emphasize the escalating economic and operational challenges faced by producers, as *Pimelea* poisoning continues to cause substantial livestock losses and financial strain across the industry. These figures underscore the unpredictability and severity of *Pimelea* outbreaks and the limitations of current management practices. The cumulative effect of these losses goes beyond the immediate economic cost; they can lead to herd destabilization, reduced breeding capacity, and an emotional toll on producers who are forced to witness the suffering and death of otherwise healthy animals.

Furthermore, these findings suggest that existing extension services, control measures, and early warning systems may not adequately address the threat. The high death toll and rising economic burden indicate an urgent need for more coordinated and science-backed management strategies. This could include improved pasture management techniques, timely identification and treatment protocols, targeted herbicide use, and development of resistant pastures. Additionally, policy-level support—such as subsidies for affected producers or investment in *Pimelea* research—may be necessary to curb the widening impact of this issue.

*Pimelea* poisoning is not just a biological challenge but also a growing socio-economic threat to cattle producers. The significant rise in financial losses, combined with high mortality rates, reinforces the urgent need for an integrated, well-funded response involving producers, researchers, government agencies, and the wider agricultural community.

### 3.4. Non-Chemical Control Methods

The survey found that 65% of producers did not attempt to control *Pimelea*, citing the ineffectiveness or high cost of available methods. Among those who implemented control measures, rotational grazing and spelling affected paddocks were the most common strategies.

Hand-weeding and slashing were reported as ineffective for large-scale control, as they can spread seeds and leave toxic plant material. Burning, while occasionally used, often leads to increased *Pimelea* seedlings in subsequent seasons [7]. Grazing management involving sheep and goats was identified as a viable alternative since these animals are less susceptible to simplexin [14]. However, establishing and managing such flocks requires significant investment in infrastructure, making it unfeasible for some producers.

### 3.5. Chemical Control and Herbicide Use

Herbicide application was reported as moderately successful by 29% of producers, with 2,4-D amine, Glyphosate, and Dicamba being the most commonly used chemicals. However, 14% of producers found herbicides ineffective, highlighting their variable efficacy. While herbicides can provide short-term control, economic feasibility and the potential impacts on non-target pasture plants remain concerns [22].

Pre-emergent herbicides combined with cropping were identified as a potential strategy, though they may lead to higher *Pimelea* populations in subsequent fallows if no additional control measures are applied [7]. Chemical control through the use of herbicides continues to be the most effective, economical, and efficient method for managing weeds in agroecosystems, making it the most widely adopted approach [23]. This is particularly relevant in the context of controlling native species like *Pimelea*, where herbicides such as tebuthiuron have demonstrated significant efficacy in both pre-emergence and post-emergence applications. The reliance on herbicides is driven by their ability to provide rapid and scalable solutions to weed infestations, which is critical for maintaining agricultural productivity and minimizing economic losses. However, the widespread use of chemical control methods also necessitates careful consideration of potential environmental impacts, such as soil health, non-target species, and herbicide resistance. Further research is needed to evaluate long-term effectiveness and develop more targeted and sustainable chemical control options.

### 3.6. Integrated Weed Management (IWM)

The survey underscored the potential of an integrated weed management (IWM) approach, combining chemical, physical, and cultural control methods. This could include using competitive pasture plants, rotational grazing, herbicide application, and targeted hand-weeding. Establishing competitive pastures can suppress *Pimelea* growth through competition, though care must be taken to prevent pasture seed contamination [7]. Integrated weed management strategies that combine chemical control with cultural, mechanical, and biological methods may offer a more sustainable approach to long-term weed management while addressing these concerns [24]. Synergistic interactions may occur among species due to modifications in resource utilization. Relative species abundances also determine the strength of an interaction. These ideas can be applied to assess the weed suppression abilities of multispecies grasslands [25].

Research on the suppressive ability of pastures against various weed species, such as parthenium weed (*Parthenium hysterophorus* L.), has demonstrated the potential for certain pasture species to effectively suppress weeds while simultaneously producing palatable biomass for livestock [26]. This dual benefit highlights the value of selecting competitive pasture species as part of an integrated weed management strategy. A recent study highlights significant differences in the ability of various grass species to suppress *Pimelea* growth. Premier digit grass (*Digitaria eriantha* Steud. var. Premier) and Rhodes grass (*Chloris gayana* Kunth.) were identified as the most effective in suppressing *Pimelea*, while Buffel grass performed the worst. This suggests that the choice of pasture species plays a critical role in managing *Pimelea* infestations [8].

Producers emphasized the importance of maintaining ground cover with trees, grass, and legumes to prevent *Pimelea* emergence. Subdividing properties into smaller paddocks for rotational grazing was another suggested strategy. However, success depends on factors such as rainfall, soil type, and infestation scale, highlighting the need for flexible and adaptive management strategies. Well-managed pastures, when stocked appropriately, could therefore serve as a key component of an improved management program for *Pimelea* control. However, the success of such an approach depends on the ability of suitable grass species to establish and persist in the harsh environmental conditions where *Pimelea* thrives. Integrating competitive pasture species into these systems could help reduce *Pimelea* density and improve overall pasture health, particularly in degraded or vulnerable areas [8]. This strategy, combined with other control methods, may offer a more sustainable and holistic solution to managing *Pimelea* infestations.

### 3.7. Ecological Role, Predation, and Traditional Knowledge of Pimelea Species

*Pimelea* species are native shrubs widespread across Australian rangelands, where they fulfil a complex ecological role. These plants often act as opportunistic colonisers of disturbed or degraded areas, particularly following events such as overgrazing, fire, or land clearing [7]. However, in contrast to many native legumes and grasses, *Pimelea* offers minimal forage value due to its low palatability and high toxicity, limiting its utility for both livestock and native herbivores.

Regarding natural herbivory, *Pimelea* species are largely avoided by grazing mammals—including cattle, sheep, kangaroos, and wallabies—due to their toxic properties. There is limited evidence of significant insect or microbial herbivory affecting *Pimelea* population dynamics, which likely contributes to their persistence and spread under high grazing pressure that suppresses more palatable species.

Despite their widespread distribution and ecological impact, there is scant formal documentation of Indigenous knowledge specifically relating to *Pimelea* species. Anecdotal reports suggest some Aboriginal groups were aware of the plants’ toxicity, particularly regarding their effects on livestock, and may have actively avoided grazing animals in heavily infested areas. However, there is no substantial evidence of traditional medicinal use or cultural significance. It is possible that traditional fire regimes and land-use practices indirectly influenced *Pimelea* prevalence, though such interactions remain poorly studied.

This highlights a critical gap in the literature and presents an opportunity for further research. Actively engaging with Indigenous communities to explore their ecological knowledge could provide valuable insights into the historical management of *Pimelea* and contribute to the development of more holistic, sustainable control strategies that integrate traditional and contemporary approaches.

### 3.8. Toxic Compound and Mechanism of Action

The toxicity associated with *Pimelea* poisoning in cattle is primarily attributed to simplexin, a potent daphnane-type orthoester diterpenoid. Simplexin is a naturally occurring phytotoxin found in several *Pimelea* species, most notably *P. trichostachya*, *P. simplex*, and *P. elongata*—species commonly associated with poisoning outbreaks in Australian rangelands [26]. It is present in various plant parts, with the highest concentrations typically found in the leaves and flowering structures.

Simplexin exerts its toxic effects by inducing vasoconstriction, particularly within the pulmonary venous system. The mechanism involves direct damage and irritation to vascular endothelial cells, leading to thickening of pulmonary vein walls, increased vascular resistance, and pulmonary hypertension. This sustained pressure impairs the normal flow of blood through the lungs, eventually resulting in right-sided congestive heart failure. Clinically, affected animals exhibit signs consistent with impaired circulation and respiratory distress, including subcutaneous oedema (commonly in the brisket), jugular vein distension, respiratory difficulty, reluctance to move, and lethargy. In some cases, skin cracking and secondary infections are observed in severely affected animals due to prolonged oedematous swelling.

The toxic effects of simplexin are exacerbated by its slow metabolism and extended persistence in the body, which can result in chronic symptoms even after exposure has ceased. Because of the irreversible vascular damage and limited ability of the animal’s body to detoxify simplexin, treatment options are minimal, and recovery depends largely on early removal from the source and supportive management [26]. This mechanism of action also helps explain why cattle, particularly in poor condition or under environmental stress, are more vulnerable to fatal outcomes following *Pimelea* ingestion. Understanding the biochemical pathway and systemic effects of simplexin is critical for developing future mitigation strategies, including targeted therapies or rumen-binding agents, though such options remain experimental at present.

Recent reviews and experimental studies provide further insight into possible mitigation strategies. Gordon [26] confirmed that no effective antidote exists for *Pimelea* poisoning and that management continues to rely heavily on early recognition and avoidance. Vaccine development has thus far been unsuccessful, and recent feeding trials investigating microbial inoculants also demonstrated limited efficacy in preventing toxic effects [27]. However, the same study reported that some absorbent materials show potential in reducing toxin absorption and may serve as a preventative option. While promising, these approaches remain under investigation, and their practical utility, cost-effectiveness, and on-farm uptake require further validation.

### 3.9. Limitations and Future Research

The survey had several limitations, including a small sample size and potential response bias, as it was conducted during a year of low *Pimelea* infestations. This may have influenced producer perceptions and management practices. Additionally, while the survey provided valuable insights into the economic and biological impacts of *Pimelea*, further research is needed to better understand the pathophysiology of *Pimelea* poisoning and to develop more effective control methods.

Addressing these research gaps through continued studies and collaboration between producers, researchers, and policymakers will be essential in improving *Pimelea* management strategies and mitigating its impact on livestock production and pasture systems.

## 4. Conclusions

The findings of this survey underscore the significant challenges posed by *Pimelea* to livestock production and pasture systems in QLD, NSW, and SA. While awareness of *Pimelea* and its impacts has increased among producers, effective management remains elusive, particularly for large-scale infestations. The cyclical nature of *Pimelea* infestations, combined with the economic and logistical challenges of control, highlights the need for a coordinated and integrated approach to management. This study also highlights the need to strengthen extension services to support producers with timely, region-specific advice and resources. Coordinated management strategies backed by collaboration among researchers, industry, and government, along with supportive policy interventions, are critical to mitigating the economic and environmental impacts of *Pimelea*. Addressing these areas will be key to enhancing the resilience and sustainability of affected agricultural systems.

## 5. Materials and Methods

### 5.1. Survey Design

The survey designed for this study consisted of 25 structured questions divided into three key sections to comprehensively assess the issue of *Pimelea*. The first section focused on the general awareness of *Pimelea*, aiming to evaluate producers’ knowledge of its species, growth stages, and ecological characteristics. The second section explored the impacts of *Pimelea* on pasture systems, livestock production, and associated financial losses, providing insights into the severity and extent of its effects. The third section examined the approaches used by producers to manage *Pimelea*, including chemical, physical, and alternative strategies such as competitive pastures and livestock diversification. These questions were carefully designed to gather detailed and actionable information (Appendix A).

### 5.2. Survey Distribution

The survey questionnaire was published online using Survey Monkey (an online survey development cloud-based software package) and runs during the months of August to October 2021 (see Appendix A). The survey was developed and delivered as both a mail survey and a face-to-face one. The Internet and mail questionnaires were promoted through several electronic forums (https://futurebeef.com.au/help-requested-pimelea-management-survey/), AgForce, QLD, and personal communication with farmers in regions known to be affected by *Pimelea.*

### 5.3. Privacy and Confidentiality of the Survey

Detailed information was provided to producers regarding the survey objectives and methods, the privacy policy to be followed, the potential outcomes, and the proposed feedback mechanism of this study. A participation consent form was also supplied at the time of providing the questionnaire. All data and result sets were password-protected and only accessible to the research team trained in confidentiality procedures. Producers’ information was de-identified and made confidential. All data were converted into an electronic form and were stored in the UQ’s e-space and password-protected. Science Low or Negligible Risk (LNR) confirms that the project “The assessment of *Pimelea* impacts on agriculture and approaches to managing *Pimelea*” (Project Number: 2021/HE000998) complies with the *National Statement on Ethical Conduct in Human Research* (2007, current revision). The University’s human research ethics committees operate in accordance with this statement to ensure ethical standards are met. Approved under Version 1.01 on 19 August 2021, this project adheres to established ethical guidelines for human research.

### 5.4. Data Analysis

Given the relatively small sample size (n = 34), the datasets were analysed on a percentage basis to identify key trends and patterns, and they can be retrieved at the following: https://www.surveymonkey.com/results/SM-WXw_2FfS_2BG0EMmB0TBN0H_2FDg_3D_3D/ (Appendix B). Aggregated results from the three sections provided valuable insights into the challenges and management practices associated with *Pimelea*. Despite the limited sample size, the findings contributed significant qualitative and quantitative data to support future research and management strategies.

## Figures and Tables

**Table 1:** Local Government Areas from which survey responses were received across the three states of Queensland, New South Wales, and South Australia.

Local Government Areas (LGAs)	State
Balonne	Queensland
Barcoo	
Western Downs	
Goondiwindi	
Maranoa	
Bourke	New South Wales
Brewarrina	
Unincorporated area of NSW	
Far North SA	South Australia
Marree region	

**Table 2:** The awareness and ability of the landholder to identify different *Pimelea* species at different growth stages, the frequency of infestation on their properties, the peak season for *Pimelea* infestations, and the dispersal method.

Question	Producers (%)
**Familiarity with *Pimelea* species**	
Yes	91
No	9
**Most common species**	
*Pimelea simplex* (desert rice-flower)	71
*Pimelea trichostachya* (flax weed)	26
*Pimelea elongata* (lakebed pimelea)	19
Don’t know	16
**Ability to identify *Pimelea* species**	
Seedling stage	37
Immature stage (not flowering)	63
Mature stage (flowering)	72
Mature stage (with seeds)	66
Dead plants (with tufts of seeds)	56
Never	6
**Frequency of *Pimelea species***	
Never	6
Very often (yearly)	47
Often (once every 2 to 5 years)	41
Rarely (<5 years)	6
**Peak season or seasons for *Pimelea***	
None	6
Summer (Dec–Feb)	29
Winter (June–Aug)	48
Spring (Sep–Nov)	65
Autumn (Mar–May)	32
≥50 mm rainfall, regardless of season	29
**How was it moved around your property**	
Wind	69
Water movement	44
Animal movement	50
Contaminated seed	0
Contaminated farm machinery	9
Contaminated vehicles	19
It was not moved around, and *Pimelea* comes up in the same areas when conditions are favorable	28
Other ways (please state)—native	13
**Where does *Pimelea* prefer to grow and establish**	
Not sure	20
On lands previously cultivated	20
On lands previously burnt	3
On heavily grazed lands	46
On soils with low fertility	30
On soils with high fertility	10
On acidic soil	7
On alkaline soil	10
Only on light red, sandy loams, and red heavy clay soils	50
No particular situation	10

**Table 3:** The agricultural impacts and activities undertaken by producers, the first notice of *Pimelea* infestation, the percentage of the property affected by *Pimelea*, the intensity of the infestation, suspected germination triggers, the density of established populations, the current situation with *Pimelea* and its impacts on pasture productivity and livestock production, the type of animals in the enterprise, and whether any of those animals were affected.

Questions	Producers (%)
**What agricultural practices do you undertake on your property?**	
Beef production	75
Sheep production	56
Goat production	47
Crop production	9
Other production	13
**The first time *Pimelea* was noticed on your property**	
Never	6
Less than 5 years ago	13
5–10 years ago	22
10–20 years ago	9
20–30 years ago	13
More than 30 years ago	31
Not sure	6
**Do you consider *Pimelea* to be a problem on your property?**	
No	6
Yes—a minor problem	31
Yes—a moderate problem	34
Yes—a major problem	28
**The percentage of land infested by *Pimelea* on your property**	
0–10%	40
10–20%	20
20–30%	7
30–40%	10
40–50%	6
More than 50%	17
**The current situation with *Pimelea***	
Nowhere	6
Along the roads/tracks	55
Within rangelands/grazing lands	77
Around the margins of lakes/ponds/rivers	10
Amongst lightly forested areas	6
On cultivated/cropping lands	3
On unproductive lands	19
Nowhere or rarely found	3
**Suspected germination triggers for *Pimelea***	
No particular event	17
High rainfall events	24
Moderate rainfall events	52
Light/low rainfall events	52
A high increase in temperature	10
A moderate increase in temperature	24
Low temperature	13
After grading tracks or soil disturbance	31
Tillage to a depth of ≥10 cm	3
Tillage to a depth of ≤10 cm	17
**The density of *Pimelea* plants established**	
0–5 plants	25
5–10 plants	28
>10 plants	31
Not sure	16
**Most affected enterprise**	
Pasture production	19
Animal production	97
Crops	0
**Affected animals**	
Sheep	13
Goats	6
Cattle	94
Horses	3
Other	3

**Table 4:** The control methods used by landholders and an indication as to whether producers attempted to control *Pimelea* and, if so, the different control methods used and the level of success achieved.

Questions	Producers (%)
**Management of *Pimelea***	
Yes	35
No	65
**Which control method used**	
Slashing	12
Uprooting/hand pulling	12
Ploughing/mechanical weeding	0
Planting forage crops or competitive pastures	6
Crash grazing young plants	6
Spell affected paddocks from cattle grazing	59
Only graze sheep or goats in *Pimelea*-affected paddocks	35
**Use of improved pasture grasses**	
Yes	23
No	77
**Impact on cattle**	
Deaths (26 per respondent)	50
Average financial loss (AUD 67,000 per producer)	43
**Aspects of management of *Pimelea***	
Cost for management per year (AUD 2100)	15
Very easy to manage	3
Easy to manage	17
Difficult to manage	50
Very difficult to manage	30

## Data Availability

The original contributions presented in this study are included in the article. Further inquiries can be directed to the corresponding authors.

## Appendix A

**Questionnaire: The assessment of *Pimelea* plants in the field and the approaches used to manage these problem plant species**
**Section A: General information**
**A1. Which local government area is your property located within?**
**A2. What agricultural practices do you undertake on your property? (mark as many boxes as applicable)**
  ☐ Beef production
  ☐ Sheep production
  ☐ Goat production
  ☐ Crop production
  ☐ Other
**Section B: Awareness of *Pimelea***
**B1. Are you familiar with the toxic *Pimelea* species?**
  ☐ Yes
  ☐ No
**B2. Which of the following species are most prevalent on your property?**
  ☐ *Pimelea simplex* (desert rice-flower)
  ☐ *Pimelea trichostachya* (flax weed)
  ☐ *Pimelea elongata* (lakebed pimelea)
  ☐ Don’t know
**B3. At which growth stage are you able to identify these species? (mark as many boxes as applicable)**
  ☐ Seedling stage
  ☐ Immature stage (not flowering)
  ☐ Mature stage (flowering)
  ☐ Mature stage (with seeds)
  ☐ Dead plants (with tufts of seeds)
  ☐ Never
**B4. When did you first notice *Pimelea* on your property?**
  ☐ Never
  ☐ Less than 5 years ago
  ☐ 5–10 years ago
  ☐ 10–20 years ago
  ☐ 20–30 years ago
  ☐ More than 30 years ago
  ☐ Not sure, I have only been managing this property for less than 5 years.
  ☐ Other
**B5. How often do these toxic *species* appear on your property?**
  ☐ Very often (yearly)
  ☐ Often (once every 2 to 5 years)
  ☐ Rarely (less than 5 years)
  ☐ Never
**B6. Which season or seasons do you most often see *Pimelea* on your property? (mark as many boxes as applicable)**
  ☐ Summer (Dec–Feb)
  ☐ Winter (June–Aug)
  ☐ Spring (Sep–Nov)
  ☐ Autumn (Mar–May)
  ☐ In response to more than 50 mm rainfall, regardless of season
  ☐ None
**B7. Which of the following events do you think trigger the germination of *Pimelea*? (mark as many boxes as applicable)**
  ☐ No particular event
  ☐ Following high rainfall events
  ☐ Following moderate rainfall events
  ☐ Following light/low rainfall events
  ☐ Following a high increase in temperature
  ☐ Following a moderate increase in temperature
  ☐ Low temperature
  ☐ After grading tracks or soil disturbance
  ☐ When a tillage implement has been used to a depth of greater than 10 cm
  ☐ When a light implement has been used to a depth of less than 10 cm
  ☐ Other
**B8. Where does *Pimelea* prefer to grow and establish? (mark as many boxes as applicable)**
  ☐ On lands previously cultivated
  ☐ On lands previously burnt
  ☐ On heavily grazed lands
  ☐ On soils with low fertility
  ☐ On soils with high fertility
  ☐ On acidic soil
  ☐ On alkaline soil
  ☐ Only on light red, sandy loams and red, heavy clay soils
  ☐ No particular situation
  ☐ Other (Please state)
**B9. If you have *Pimelea*, how do you think it’s seed is moved around your property?**
  ☐ Wind
  ☐ Water movement (irrigation, rainfall, overland water movement)
  ☐ Animal movement (domesticated and wildlife)
  ☐ Contaminated seed used in pasture improvement and forage crops
  ☐ Contaminated farm machinery
  ☐ Contaminated vehicles
  ☐ It is not moved around and *Pimelea* comes up in the same areas when conditions are favourable
**B10. Currently in 2021, where is *Pimelea* found on your property? (mark as many boxes as applicable)**
  ☐ Along the roads/tracks
  ☐ Within rangelands/grazing lands
  ☐ Around the margins of lakes/ponds/rivers
  ☐ Amongst lightly forested areas
  ☐ On cultivated/cropping lands
  ☐ On unproductive lands
  ☐ Nowhere or rarely found
**Section C: Impacts of *Pimelea* in pasture**
**C1. Do you consider *Pimelea* to be a problem on your property?**
  ☐ No
  ☐ Yes—a minor problem
  ☐ Yes—a moderate problem
  ☐ Yes—a major problem
**C2. What percentage of your property is infested with *Pimelea* in years that favour its establishment?**
  ☐ 0–10%
  ☐ 10–20%
  ☐ 20–30%
  ☐ 30–40%
  ☐ 40–50%
  ☐ More than 50%
**C3. In years when infestations occur on your property, what do you think is the average density of *Pimelea* plants found in a 1 m^2^ area?**
  ☐ 0–5 plants
  ☐ 5–10 plants
  ☐ more than 10 plants
  ☐ Not sure
**C4. Which enterprise is most affected on your property by *Pimelea?* (mark as many boxes as apply)**
  ☐ Pasture production (and therefore animal production)
  ☐ Animal production (due to effects on animal health)
  ☐ Crops
  ☐ Other
**C5. What types of animals are affected on your property? (mark as many boxes as apply)**
  ☐ Sheep
  ☐ Goats
  ☐ Cattle
  ☐ Horses
  ☐ Other please specify
**C6. In years when *Pimelea* is prevalent, what is an average mortality rate and what is the estimated financial impact from reduced animal production, providing quality fodder and/or agistment and death of severely affected animals?**
  ☐ Death (Number of animals)
  ☐ Annual financial impact (dollars)
  ☐ Extra labour time in caring for affected animals (days per year)
**Section D: Control of *Pimelea* in pasture**
**D1. Have you tried to control *Pimelea* on your property?**
  ☐ Yes
  ☐ No
**D2. If yes, which method did you use? (mark as many boxes as applicable)**
  ☐ Slashing
  ☐ Uprooting/hand pulling
  ☐ Ploughing/mechanical weeding
  ☐ Planting forage crops or competitive pastures
  ☐ Crash grazing young plants
  ☐ Chemical (specify which ones)
  ☐ Spell affected paddocks from cattle grazing
  ☐ Only graze sheep or goats in *Pimelea*-affected paddocks
  ☐ Other techniques (specify)
**D3. If yes, which individual or combination of methods have worked the best for you?**
  Please specify:
  1. --------------------------------------------------------------------------------
  2. --------------------------------------------------------------------------------
  3. --------------------------------------------------------------------------------
**D4. Have you ever tried growing improved pastures grasses to help suppress and control *Pimelea*?**
  ☐ Yes
  ☐ No
**D5. If yes, name the improved pastures grasses used and indicate if this was a successful approach or not.**
**D6. How much do you spend on your property to control *Pimelea per annum*? (Dollars or days per years).**
**D7. Overall, what are your thoughts about control of *Pimelea* in pasture?**
  ☐ Very easy to manage
  ☐ Easy to manage
  ☐ Difficult to manage
  ☐ Very difficult to manage
Thank you for participating in this survey. Once the data is analysed, the outcomes will be made available to all who request copies. If you would like to receive a summary of the key findings about pasture management practices, please fill in your details below.

## Appendix B

https://www.surveymonkey.com/results/SM-WXw_2FfS_2BG0EMmB0TBN0H_2FDg_3D_3D/ (accessed date: 20 August 2021)
